# Endocan as a biomarker for acute respiratory distress syndrome: A systematic review and meta‐analysis

**DOI:** 10.1002/hsr2.70044

**Published:** 2024-09-02

**Authors:** Amir Hossein Behnoush, Amirmohammad Khalaji, Hoomaan Ghasemi, Ghazaal Alavi Tabatabaei, Amirsaeed Samavarchitehrani, Zahra Vaziri, Morvarid Najafi, Mitra Norouzi, Elina Ghondaghsaz, Elahe Amini, Alexandre Gaudet

**Affiliations:** ^1^ School of Medicine Tehran University of Medical Sciences Tehran Iran; ^2^ Center for Orthopedic Trans‐Disciplinary Applied Research Tehran University of Medical Sciences Tehran Iran; ^3^ Isfahan Cardiovascular Research Center, Cardiovascular Research Institute Isfahan University of Medical Sciences Isfahan Iran; ^4^ Islamic Azad University Tehran Faculty of Medicine Tehran Iran; ^5^ Student Research Committee Babol University of Medical Sciences Babol Iran; ^6^ Faculty of Life Sciences and Biotechnology Shahid Beheshti University Tehran Iran; ^7^ Undergraduate Program in Neuroscience University of British Columbia Vancouver British Columbia Canada; ^8^ Tehran Medical Sciences Branch Islamic Azad University Tehran Iran; ^9^ Univ. Lille, CNRS, Inserm, CHU Lille, Institut Pasteur de Lille, U1019‐UMR9017‐CIIL‐Centre d'Infection et d'Immunité de Lille, CHU Lille Pôle de Médecine Intensive‐Réanimation Lille France

**Keywords:** ARDS, biomarker, endocan, meta‐analysis, systematic review

## Abstract

**Background and Aims:**

Endocan is a marker of endothelial damage. Data regarding the association of this proteoglycan and acute respiratory distress syndrome (ARDS) is discrepant. Hence, this study sought to investigate the possible correlation between serum/plasma endocan concentration and ARDS.

**Methods:**

A systematic review and meta‐analysis of international online databases was conducted following PRISMA guidelines. PubMed, SCOPUS, Embase, and Web of Science were searched in March 2023, with the leading search terms being “ARDS” OR “respiratory distress” AND “endocan” and other associated terms. Studies that measured endocan levels in patients with ARDS and compared it with non‐ARDS controls or within different severities of ARDS were included. We performed a random‐effect meta‐analysis for pooling the differences using standardized mean difference (SMD) and 95% confidence interval (CI).

**Results:**

We included 14 studies involving 1,058 patients. Those developing ARDS had significantly higher levels of endocan compared to those without ARDS (SMD: 0.47, 95% CI: 0.10–0.84, *p* = 0.01). Our meta‐analysis of three studies found that endocan levels in ARDS nonsurvivors were significantly higher than in survivors (SMD: 0.31, 95% CI: 0.02–0.60, *p* = 0.03). Three studies investigated endocan levels in different severities of ARDS. Only one of these studies reported significantly higher endocan levels in patients with worsening acute respiratory failure at Day 15. The other two reported no significant association between ARDS severity and circulating endocan levels.

**Conclusion:**

Blood endocan levels were significantly higher in patients with ARDS than those without. Additionally, among patients with ARDS, blood endocan values were significantly elevated in nonsurvivors compared to survivors. These findings could help researchers design future studies and solidify these findings and finally, clinicians to take advantage of measuring endocan in clinical settings for assessment of patients with ARDS.

## 1 INTRODUCTION

Acute respiratory distress syndrome (ARDS) is defined as bilateral diffuse infiltrates on chest radiography and non‐cardiogenic respiratory failure with an acute beginning, leading to a defect in oxygen saturation of red blood cells, based on the updated Berlin definition.^1^ From the pathophysiological aspect, it is characterized by fluid collection in the alveolar space, causing alveolar edema. Changes in the microvascular endothelium play an essential role in the acute inflammatory response. To realize this, the endothelium becomes inflamed and leaky, allowing innate immune cells to cross the barrier to the infection site.^2^ The progression of this phenomenon leads to ARDS, which is a result of indirect (sepsis and multiple trauma) or direct (mostly pneumonia) damage to the lung. This is why pneumonia and sepsis are the most common causes of ARDS.^3^ ARDS represents about 5% of hospitalized patients needing mechanical ventilation, with an incidence of 15–70 cases per 100,000 individuals per year, based on findings of a large global study.^4^ So far, the role of many cells and molecules in ARDS has been known, and they are potent diagnostic, prognostic, and therapeutic implications of this disease. However, many of them have not been studied enough and used in clinical settings. Some might be hard to measure and some could not have high prognostic value. In this regard, there is a need for novel biomarkers for ARDS as a life‐threatening condition.

Endocan, also named Endothelial cell‐specific molecule 1 (ESM‐1), is a dermatan sulfate proteoglycan.^5^ The main secreted endocan is from pulmonary and renal endothelial cells upon cytokine stimulation. Therefore, it can be considered an endothelial cell damage marker. Moreover, endocan performs a function in inflammatory diseases as they are endothelial‐dependent in many cases.^6^, ^7^ As an endothelial factor, the change of endocan has been reported in several infectious and noncommunicable diseases, adding to its clinical utility.^8^, ^9^, ^10^, ^11^, ^12^, ^13^, ^14^ Evidence suggests altering levels of endocan in patients with ARDS, pneumonia, and pulmonary thromboembolism showing that endocan is involved in the vascular damage molecular mechanisms in the respiratory system.^11^, ^15^

Specifically, studies have measured circulating levels of endocan in ARDS patients showing contradictory results since some studies have found a significant relationship between endocan levels while others indicated no considerable difference. Some studies have also suggested differences in endocan levels among survivors and nonsurvivors of ARDS, investigation of which could have high clinical value. The aim of this study was to conduct a systematic review and meta‐analysis of original studies on the current literature to explore the relationship between circulating endocan levels and ARDS. Results of the current study could help clinicians and researchers understand the utilities of this novel biomarker and focus on them with further research and probably take advantage of it in clinical settings.

## 2 METHODS

### 2.1 Search strategy

Based on Preferred Reporting Items for Systematic Reviews and Meta‐Analyses (PRISMA) guidelines,^16^ this systematic review was conducted on four international online databases, including PubMed, SCOPUS, Embase, and Web of Sciences on March 24, 2023, without any filters or limitations. Our selected search terms were “ARDS” OR “respiratory distress” AND “endocan” and other related terms which are present in Supporting Information S1: Table 1. AK and AHB reviewed all the studies starting with the title and abstract for inclusion independently and then continued with reviewing the full text of selected studies primarily. In addition, relevant websites (e.g., Researchgate and Google Scholar) and references used in the included studies were screened for probable other related studies. Due to the review nature of systematic review and meta‐analysis, there was no need for ethics committee approval and informed consent.

### 2.2 Screening, study selection, and data extraction

The inclusion criteria in our study selection contained clinical studies: (1) which measured circulating levels of endocan in ARDS cases and controls OR (2) reported endocan levels in different causes of respiratory distress cases OR (3) about correlations between endocan levels and severity of ARDS OR (4) reported endocan levels in ARDS survivors and nonsurvivors OR (5) endocan levels as a diagnostic and/or prognostic marker of ARDS. Exclusion criteria were studies: (1) which did not report endocan levels; (2) conference abstracts, review articles, case reports, or case series; (3) which did not consider ARDS. The PICO (Population, Intervention, Comparison, and Outcome) for our study was: P: ARDS cases, I: plasma or serum levels of endocan as a diagnostic or prognostic marker in ARDS, C: healthy controls, and O: endocan levels between ARDS and controls in addition to survivors and nonsurvivors of ARDS.

### 2.3 Data extraction and quality assessment

Data extraction was done by two researchers (AK and AHB) separately. Our extracted data contained: (1) first author, country and year of publication, and study design; (2) population of all patients, and population of each group (patients and control) separately; (3) mean age ± standard deviation (SD); (4) male percentage of total patients; (5) time of endocan measurement; (6) estimated diagnostic and prognostic values of endocan; and (7) main findings. In case of a lack of sufficient data for extraction and analysis, the corresponding authors of the included studies were contacted to provide data.

We used the Newcastle‐Ottawa Scale (NOS) for quality assessment of the included studies.^17^ Two researchers (AK and AHB) assessed the risk of bias in the included studies. In NOS, selection, comparability, and outcome are used for the evaluation of study quality and a total NOS score ≥ 8 reveals that the study is high quality.

### 2.4 Statistical analysis and data synthesis

All statistical analyses were performed adhering to relevant recommendations for analysis, reporting, and interpretation of clinical research^18^ and “Statistical Analyses and Methods in the Published Literature” (SAMPL) guidelines for reporting statistical analyses.^19^ Our data synthesis was performed using mean and SD of endocan levels in ARDS patients and in both survivors and nonsurvivors of the disease. Our meta‐analysis was performed on levels of endocan in (1) ARDS cases in comparison to controls; and (2) survivors and nonsurvivors of ARDS. Random‐effect meta‐analysis was conducted to measure the pooled effect size using standardized mean difference (SMD) with its 95% confidence interval (CI). The heterogenicity of studies was evaluated by *Q* and Higgin's *I* ^2^. Heterogenicity of ≤25% was known to be low, range of 26%–75% moderate, and >75% high.^20^ Since some of the included studies reported endocan levels as the median and interquartile range (IQR), methods suggested by Luo et al.^21^ and Wan et al.^22^ were used to convert them to mean and standard deviation (SD). All analyses were performed using STATA (version 17, Stata Corp.) with a *p* of <0.05 as the statistical significance cutoff.

## 3 RESULTS

### 3.1 Literature search and included study characteristics

**Figure 1:**
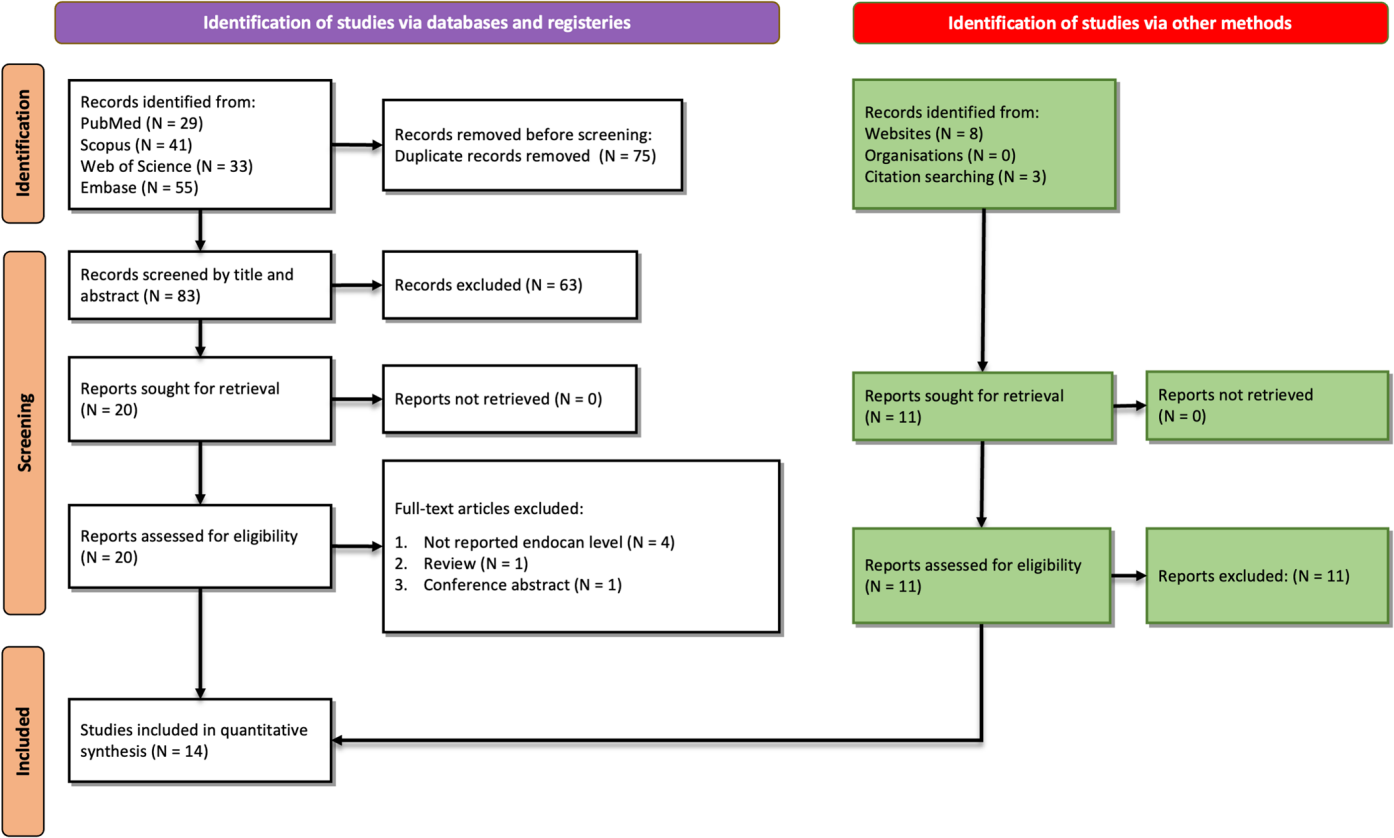
PRISMA flowchart for search and screening of studies.

The initial search resulted in 158 studies, including 29 from PubMed, 41 from Scopus, 55 from Embase, and 33 from Web of Science. After the removal of duplicates (*n* = 75), a total of 83 studies were screened based on their title/abstract after which 20 studies remained. Screening of the full text of these studies based on inclusion/exclusion criteria resulted in the removal of six other studies. Details of the search flow and reasons for exclusion are shown in Figure 1.

Finally, a total of 14 studies were included in our systematic review.^23^, ^24^, ^25^, ^26^, ^27^, ^28^, ^29^, ^30^, ^31^, ^32^, ^33^, ^34^, ^35^, ^36^ Table 1 illustrates the characteristics of the included studies. Seven of the studies were conducted in France,^24^, ^28^, ^29^, ^30^, ^34^, ^35^, ^36^ while two were in the United States^32^, ^33^ and two were in Greece.^25^, ^27^ The three studies by Gaudet et al.^28^, ^29^, ^30^ investigated the same population, so the studies assessed 1058 individual patients, details of each are shown in Table 1. Among these 14 studies, 11 had prospective design and 3 had retrospective design. The mean age of patients was 63.49 ± 14.98 years and 59.54% were male. Moreover, all studies had high quality based on the NOS quality assessment score (Supporting Information S1: Table 2). As is evident from this table, all studies scored 7 in these criteria, representing high quality. However, none of the studies could gain a score from the “comparability” domain, highlighting the shortcomings of these studies.

**Table 1:** Baseline characteristics of included studies.

Author	Year	Design	Location	Specimen	Timing of endocan measurement	Population	*N*, Total	Mean age	Male (%)	Main findings
Tang et al.	2014	Prospective cohort	China	Plasma	At admission up to 24 h	Patients with ARDS due to pulmonary infection, sepsis, aspiration, and blood transfusion	42	68 ± 15	64.3%	In the ARDS group, there was a strong correlation between endocan levels and the APACHE II score (*r* = 0.676, *p* < 0.001, *n* = 42). Endocan levels were greater in nonsurvivors than in survivors (median (IQR) 5.01 (2.98–8.44) vs. 3.01 (2.36–4.36) ng/mL, respectively; *p* = 0.017). Endocan can independently predict ARDS mortality with a hazard ratio of 1.374 (95% CI: 1.150–1.641).
Palud et al.	2015	Prospective observational pilot study	France	Plasma	At baseline	Patients presenting with septic shock	20	61 [54–68]	70%	Plasma endocan was lower in the septic shock patients who developed respiratory failure on Day 3 compared to patients who did not (1.9 [0.99–3.1] vs. 5.2 [3.1–17.2] ng/mL; *p* = 0.032).
Tsangaris et al.	2017	Prospective observational study	Greece	Plasma	At admission	ARDS patients in ICU	53	64.13 ± 15.4	62%	Endocan >13 ng/mL was associated with unassisted ventilation during 28 days of follow‐up (OR: 0.09, 95% CI: 0.01–0.79; *p* = 0.03).
Orbegozo et al.	2017	Prospective cohort	Belgium	Plasma	At the time of diagnosis and the next day morning	ARDS patients admitted to ICU	37	61 ± 17	67%	Higher endocan concentration was observed in poor evolution patients than good evolution in T1 (the following morning of ARDS diagnosis) 12.0 (6.8–18.6) versus 7.2 (5.4–12.5), *p* < 0.01]. Additionally, a cutoff point of endocan of 6 and 14 ng/mL at T0 and T1 have good sensitivity and specificity for excluding poor prognosis, respectively.
Ioakeimidou et al.	2017	Prospective cohort	Greece	Serum	At baseline and 24 h later	ICU patients with positive SIRS and one of the following infections: acute pyelonephritis, community‐acquired pneumonia, acute intraabdominal infection, primary bacteremia, or ventilator‐associated pneumonia	175	66.8 ± 18.4	63.4%	Any rise in endocan from the initial value was independently linked to the development of ARDS OR of 16.65 (95% CI: 4.85–57.11, *p* < 0.001).
Gaudet et al.	2018	Prospective cohort	France	Plasma	At enrollment	Patients who were diagnosed with severe sepsis or septic shock within 24 h of admission in ICU	72	63 [53–78]	56%	Endocan values higher than 5.36 ng/mL at baseline were associated with the absence of progression to ARDS after 72 h (OR: 0.001, 95% CI: 0–0.215, *p* = 0.011). On the other hand, the cutoff point of 2.54 ng/dL has high specificity for predicting ARDS at 72 h (Sp = 1, 95% CI: 0.94–1).
Gaudet et al.	2019	Prospective cohort	France	Plasma	At enrollment and 73 h later	Patients who were diagnosed with severe sepsis or septic shock within 24 h of admission in ICU	72	63 [53–78]	56%	Endocan levels in patients without ARDS steadily dropped throughout the course of the 72‐h period after enrollment, with median [IQR] values dropping from 9.2 [5.6–14.8] ng/mL at enrollment to 3.9 [2.6–7.7] ng/mL 72 h later. Endocan levels in patients who developed mild ARDS gradually increased from 2.5 [1.3–3.4] ng/mL at enrollment to 4.1 [2.3‐7.3] ng/mL at 72 h. Patients who developed moderate or severe ARDS showed a greater rise in blood endocan, with median [IQR] levels increasing from 4.7 [2.5–5.4] ng/mL at enrollment to 11 [9.5–12.6] ng/mL at 72 h.
Gaudet et al.	2019	Prospective cohort	France	Plasma	Time of ARDS diagnosis and 24 h later	ARDS patients	39	59 [44–68]	72%	Endocan cleavage ratio (ECR) was significantly different between HIP (−7% [− 19% to − 5%]) and NHIP groups (6% [−3% to 16%]). (*p* < 0.01). to show clinically relevant diagnostic values (AUC = 0.84, 95% CI: 0.69–0.94, *p* < 0.01)
Gaudet et al.	2022	Prospective cohort	France	Plasma	Time of ICU admission	Patients with acute respiratory failure from COVID‐19	82	64.89 ± 11.7	77.2%	Endocan was significantly higher in patients with acute respiratory failure worsening at day 15, compared to those who did not (9.13 ± 15.34 vs. 3.39 ± 3.08, *p* < 0.01).
Ying et al.	2019	Prospective cohort	China	Plasma	At admission	Patients diagnosed with severe pneumonia and admitted to the respiratory ICU	145	57.65 ± 10.1	51%	Plasma endocan was recognized as the independent risk factor for developing ARDS in patients (OR: 1.57, 95% CI: 1.14–2.25, *p* = 0.021). Plasma endocan resulted in an AUC of 0.754, 95% CI of 0.642–0.866, a cutoff value of 11.6 ng/mL, a sensitivity of 78.7%, and a specificity of 70.3%, respectively (*p* < 0.01).
Whitney et al.	2020	Retrospective cohort	United States	Plasma	Within 72 h of sepsis diagnosis, Days 3–6 and 7–14	Patients younger than 18 years old with sepsis from extrapulmonary source or without ARDS and control group	119	NR	48.7%	A higher endocan level was associated with a higher risk for complicated courses in the septic patients (AUC: 0.81, 95% CI: 0.72–0.89).
Yu et al.	2021	Prospective cohort	United States	Plasma	Within 24 h of ICU admission	ICU patients with severe sepsis and acute respiratory failure	228	NR	51.3%	Severe AKI patients in this study had higher levels of plasma endocan compared to no severe AKI group (150.9 ng/mL, IQR 83.7–347.1 ng/mL vs. 94.7 ng/mL, IQR 38.4–205.8 ng/mL, *p* < 0.001). Also, according to the ROC curve, endocan had moderate discriminative powers for severe AKI (AUC = 0.61, 95% CI: 0.54–0.69, *p* = 0.004).
Pascreau et al.	2021	Retrospective cohort	France	Plasma	At admission, Days 1–2, 3–4, and 5–6	Patients with COVID‐19 pneumonia	74	64 [55–71]	80%	Patients with COVID‐19 had higher levels of endocan at baseline compared to the control group (3.4 ng/mL [IQR: 1.8–7.5] vs. 1.6 ng/mL [IQR: 1.0–2.1], respectively, *p* = 0.0031). However, there was no significant difference between patients who progressed ARDS and patients who did not 3.7 [2.8–9.6] ng/mL vs. 3.2 [1.5–5.7] ng/mL, respectively, *p* = 0.2231). Endocan levels significantly increased in patients with mild/moderate ARDS at the 3–4 (*p* = 0.0084) and 5–6 days (*p* = 0.0107), whereas patients with severe ARDS had no increase.
Levy et al.	2023	Retrospective cohort	France	Plasma	Days 0 and 7	COVID‐19 patients undergoing V‐V ECMO implantation	11	NR	81.8%	Nonsurvivors had higher endocan at Day 7 (median [IQR] = 12.9 [6.1–19] ng/mL vs. 4.3 [3.9–5.6] ng/mL; *p* = 0.017) and a larger increase in the endocan level (median [IQR] = + 208 [−1 to +612] % vs. −49 [−65 to +52] %; *p* = 0.03). According to the results, a large drop in plasma endocan assessed 1 week after ECMO implantation would be associated with patient survival upon ICU discharge among those with V‐V ECMO.

*Note*: Data are presented as mean ± standard deviation or median [interquartile range] or percentage.

Abbreviations: AKI, acute kidney injury; APACHE II, Acute Physiology and Chronic Health Evaluation II; ARDS, acute respiratory distress syndrome; AUC, area under the curve; CI, confidence interval; COVID‐19, coronavirus disease of 2019; ECMO, extracorporeal membrane oxygenation; HIP, hyperinflammatory phenotype; ICU, intensive care unit; IQR, interquartile range; NHIP, non‐hyperinflammatory phenotype; NR, not reported; OR, odds ratio; SIRS, systemic inflammatory response syndrome.

### 3.2 Meta‐analysis of endocan levels in patients with and without ARDS

**Figure 2:**
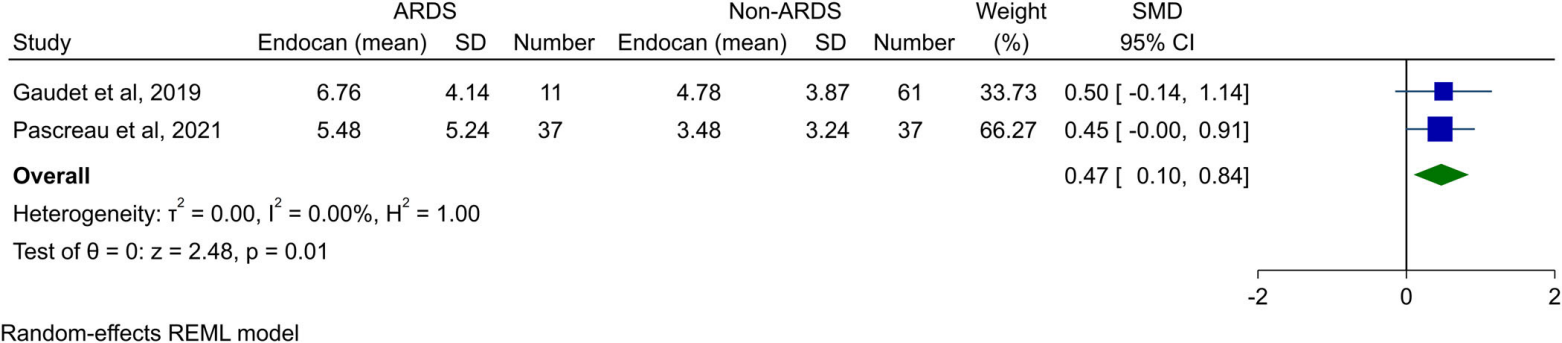
Forest plot showing meta‐analysis of endocan levels in comparison of patients with and without ARDS. ARDS, acute respiratory distress syndrome; CI, confidence interval; SD, standard deviation; SMD, standardized mean difference.

Random‐effect meta‐analysis of two studies^29^, ^34^ for comparison of patients with ARDS versus controls showed that patients developing ARDS had significantly higher levels of endocan, compared to those without (SMD: 0.47, 95% CI: 0.10–0.84, *p* = 0.01, Figure 2). There was no heterogeneity observed.

### 3.3 Meta‐analysis of endocan levels in ARDS patients who died versus survived

**Figure 3:**
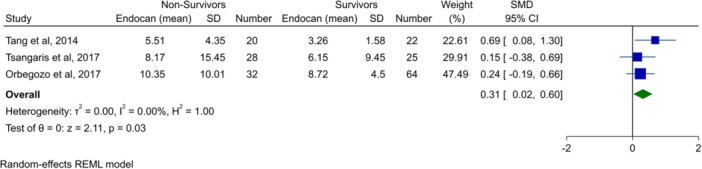
Forest plot showing meta‐analysis of endocan levels in comparison of ARDS patients who died versus survivors. ARDS, acute respiratory distress syndrome; CI, confidence interval; SD, standard deviation; SMD, standardized mean difference.

Three studies investigated endocan levels in patients with ARDS and compared the groups who died and survivors.^23^, ^25^, ^26^ Results indicated that ARDS patients who died had significantly higher endocan levels (SMD: 0.31, 95% CI: 0.02–0.60, *p* = 0.03, Figure 3) with no heterogeneity.

### 3.4 Endocan levels in different severities of ARDS

Three studies evaluated circulating endocan in ARDS severities.^23^, ^34^, ^36^ Tang et al.^23^ compared moderate (*n* = 23) and severe (*n* = 19) ARDS in terms of blood endocan levels. They found no difference in blood endocan levels (severe: 4.35 [2.97–7.28] ng/ml vs. moderate: 3.21 [2.38–4.96] ng/mL, *p* = 0.176). Gaudet et al.^36^ assessed endocan levels in acute respiratory failure (ARF) patients from COVID‐19 and compared patients who had worsening ARF needing secondary intubation with patients without worsening ARF. It was shown that patients with worsening ARF at day 15 had significantly higher blood endocan levels (9.13 ± 15.34 vs. 3.39 ± 3.08, *p* < 0.01) compared to those without worsening ARF. Finally, Pascreau et al.^34^ found no significant difference in endocan levels between different severities of ARDS (mild‐to‐moderate vs. severe ARDS).

## 4 DISCUSSION

To the best of our knowledge, this is the first systematic review and meta‐analysis investigating the correlation between blood endocan profile and ARDS. The main findings of the present study indicated significantly higher plasma endocan concentrations in patients with ARDS compared to those without. Additionally, plasma endocan values were significantly elevated in ARDS patients who died in comparison to those who survived.

There are discrepancies in the literature regarding the endocan values in patients with ARDS compared to those without. Our study revealed that individuals who developed ARDS had significantly higher plasma endocan levels than those without ARDS. Consistent with our results, Gaudet et al. assessed blood endocan levels of 72 septic patients without ARDS on baseline over 72 h. Eleven of them progressed to ARDS within 72 h following inclusion. They observed a continuous decrease in endocan value among patients without ARDS, whereas endocan levels were increased in those who developed ARDS.^29^ To the contrary, a retrospective cohort of 74 subjects with COVID‐19 pneumonia conducted by Pascreau et al. reported no significant difference in endocan levels between patients who progressed into ARDS and those who did not.^34^ Additionally, previous meta‐analyses have shown that endocan‐1 and syndecan‐1 were elevated in patients with COVID‐19.^11^, ^37^ To elucidate the different results of the previous studies, several hypotheses could be suggested, including different settings of endocan measurement, different predisposing conditions, and differences in control groups. For instance, the results of Pascreau et al.^31^, ^34^ were based on endocan levels at admission when patients had not developed ARDS yet. In contrast, Gaudet et al.^29^ analyzed plasma endocan values at the time of diagnosis and at 12, 24, 48, and 72 h following inclusion. Furthermore, in the study of Gaudet et al.,^29^ the predisposing condition was sepsis, while in the study conducted by Pascreau et al.,^31^, ^34^ the predisposing condition was COVID‐19 pneumonia. Moreover, the control subjects in the Pascreau et al.^34^ study were healthy hospital staff with no underlying diseases, contrary to the study of Gaudet et al.,^29^ where patients with sepsis served as the control group.

Our results showed significantly elevated endocan concentration among ARDS patients who died compared to those who survived. In line with our findings, in a prospective cohort of 42 patients with ARDS, endocan levels measured on Day 1 after ARDS diagnosis were significantly elevated in nonsurvivors compared to survivors. Hence, these data support the hypothesis that endocan is a promising biomarker for predicting severity and mortality in ARDS patients.^23^ Additionally, a prospective cohort of 37 patients who had developed ARDS and were admitted to the ICU showed significantly higher endocan levels 1 day after ARDS diagnosis in patients who expired or needed more than 10 days of mechanical ventilation compared to survivors with less than 10 days of ventilator support, suggesting endocan a useful biomarker of ARDS severity.^26^ On the other hand, a prospective observational study on 53 subjects with ARDS in the ICU reported no significant difference in plasma endocan value between nonsurvivors and survivors.^25^ These inconsistencies in the conclusions across previous studies might be due to patient status differences. For instance, in the studies of Tang et al. and Orbegozo et al.,^23^, ^26^ nonsurvivors had significantly higher APACHE II scores compared to survivors, while in the study by Tsangaris et al.,^25^ no significant difference in APACHE II scores was observed between nonsurvivors and survivors.

Concerning blood endocan values among patients with different ARDS severities, Pascreau et al. reported no significant difference in endocan concentration among COVID‐related ARDS subjects with different severities.^34^ Consistent with these results, Tang et al. compared endocan plasma levels between moderate and severe ARDS cases and found no difference.^23^ Conversely, Gaudet et al.^36^ indicated that among individuals with ARF due to COVID‐19, those with late ARF worsening had significantly higher blood endocan values than those without. It appears that differences in predisposing conditions and ARDS severities in the previous studies could be potential explanations for the inconsistent results mentioned earlier.

Our findings in this systematic review and meta‐analysis have several clinical and research implications. Higher circulatory endocan levels in patients with ARDS could help clinicians not only in diagnosis but also in predicting the prognosis of those with ARDS. Further large‐scale studies are needed to confirm these findings. Researchers should focus on the determination of the prognostic ability of endocan by calculation of its sensitivity, specificity, and area under the curve (AUC). Moreover, the association between endothelial dysfunction and ARDS could be further explored for which biomarkers such as endocan‐1 and syndecan‐1 have high value.

## 5 STRENGTHS AND LIMITATIONS

This was the first study that pooled the data for the association of endocan levels and ARDS. Higher levels of endocan in patients with ARDS had clinical and research applications. By further research on this blood biomarker, researchers could pave the way for future use of this biomarker in clinics. Assessment of endocan in different clinical settings and countries is another strength of this manuscript. Showing its prognostic ability in patients with ARDS by comparing endocan levels in survivors and nonsurvivors is another strength discussed by our investigation.

The current study had some limitations that should be taken into account when interpreting the findings. First, it is possible to overlook some pertinent publications even with a thorough search strategy across electronic databases, reviewing relevant websites, and going back and forth in citations. Second, since the compared groups in the pertinent studies had varied ARDS severities from one another, performing a meta‐analysis related to plasma endocan among ARDS patients with different severities was not possible. Third, bias might result from the limited number of included studies, the small sample sizes, and the substantial heterogeneity among them. Similarly, since additional analyses, such as assessment of publication bias, have low power in analyses comprised of less than 10 studies, we were unable to perform them. Fourth, while regional factors and trends may have an impact on the geographic distribution of the included studies, the findings cannot be generalized to all patients globally. Finally, different settings of endocan measurements might contribute to contrasting results, limiting the generalizability of our findings.

## 6 CONCLUSION

In conclusion, blood endocan concentrations were significantly higher in patients with ARDS than those without. Additionally, among patients with ARDS, blood endocan values were significantly elevated in nonsurvivors compared to survivors. Further large‐scale cohort studies are warranted to explore the potential connections more comprehensively.

## AUTHOR CONTRIBUTIONS

**Amir Hossein Behnoush**: Writing—original draft; conceptualization; formal analysis; visualization. **Amirmohammad Khalaji**: Supervision; conceptualization; writing—original draft; writing—review and editing. **Hoomaan Ghasemi**: Data curation; writing—original draft. **Ghazaal Alavi Tabatabaei**: Data curation; writing—original draft. **Amirsaeed Samavarchitehrani**: Writing—original draft; data curation. **Zahra Vaziri**: Data curation; writing—original draft. **Morvarid Najafi**: Data curation; writing—original draft. **Mitra Norouzi**: Data curation; writing—original draft; writing—review and editing. **Elina Ghondaghsaz**: Data curation; writing—original draft; writing—review and editing. **Elahe Amini**: Data curation; writing—original draft. **Alexandre Gaudet**: Writing—review and editing.

## CONFLICT OF INTEREST STATEMENT

The authors declare no conflict of interest.

## TRANSPARENCY STATEMENT

The lead author Amirmohammad Khalaji affirms that this manuscript is an honest, accurate, and transparent account of the study being reported; that no important aspects of the study have been omitted; and that any discrepancies from the study as planned (and, if relevant, registered) have been explained.

## Supporting information

Supporting information.

## Data Availability

All data generated or analyzed during this study are included in this published article and its supplementary information files.
