# Supplementary material for: Endocan as a biomarker for acute respiratory distress syndrome: A systematic review and meta‐analysis
Source: Health Sci Rep. 2024 Sep 2;7(9):e70044. doi: 10.1002/hsr2.70044 (PMC11368821; doi:10.1002/hsr2.70044)
Supplement: Supplementary file 1 — Supporting information. [file HSR2-7-e70044-s001.docx]

***Supplementary Materials***

***Supplementary Table 1.*** *Search strategy for each database*

| **Query** | | **Results**  **(March 24, 2023)** |
| --- | --- | --- |
| ***PubMed*** | | |
| #1 | (“ARDS” OR “respiratory distress*” OR “acute respirat*” OR "Respiratory Distress Syndrome"[Mesh] OR "Respiratory Distress Syndrome" OR “shock lung”) | 138,570 |
| #2 | ("Endocan" OR "ESM-1" OR "ESM1" OR "ESM 1" OR "endothelial cell-specific molecule 1" OR "ESM1 protein, human" [Supplementary Concept]) | 726 |
| **#3** | **#1 AND #2** | **29** |
| ***SCOPUS*** | | |
| #1 | TITLE-ABS-KEY(“ARDS” OR “respiratory distress*” OR “acute respirat*” OR "Respiratory Distress Syndrome" OR "Respiratory Distress Syndrome" OR “shock lung”) | 242,791 |
| #2 | TITLE-ABS-KEY("Endocan" OR "ESM-1" OR "ESM1" OR "ESM 1" OR "endothelial cell-specific molecule 1" OR "ESM1 protein, human") | 971 |
| **#3** | **#1 AND #2** | **41** |
| ***Embase*** | | |
| #1 | (“ARDS” OR “respiratory distress*” OR “acute respirat*” OR "Respiratory Distress Syndrome" OR "Respiratory Distress Syndrome" OR “shock lung”) | 296,821 |
| #2 | ("Endocan" OR "ESM-1" OR "ESM1" OR "ESM 1" OR "endothelial cell-specific molecule 1" OR "ESM1 protein, human") | 1,099 |
| **#3** | **#1 AND #2** | **55** |
| ***Web Of Science*** | | |
| #1 | TS=(“ARDS” OR “respiratory distress*” OR “acute respirat*” OR "Respiratory Distress Syndrome" OR "Respiratory Distress Syndrome" OR “shock lung”) | **118,508** |
| #2 | TS=("Endocan" OR "ESM-1" OR "ESM1" OR "ESM 1" OR "endothelial cell-specific molecule 1" OR "ESM1 protein, human") | 931 |
| **#3** | **#1 AND #2** | **33** |

Total: 158

After removing duplicates: 83

***Supplementary Table 2****. Quality Assessment based on the Newcastle-Ottawa Scale (NOS) manual for cohort studies*

| **Study**  **(Year)** | **Selection** | | | | **Comparability** | **Outcome** | | | **Overall**  **Score** |
| --- | --- | --- | --- | --- | --- | --- | --- | --- | --- |
|  | **Representativeness** | **Control** | **Exposure** | **Outcome** |  | **Outcome** | **Follow-up** | **Lost to Follow-up** |  |
| Tang et al. (2014) | * | * | * | * | - | * | * | * | 7 |
| Palud et al. (2015) | * | * | * | * | - | * | * | * | 7 |
| Tsangaris et al. (2017) | * | * | * | * | - | * | * | * | 7 |
| Orbegozo et al. (2017) | * | * | * | * | - | * | * | * | 7 |
| Ioakeimidou et al. (2017) | * | * | * | * | - | * | * | * | 7 |
| Gaudet et al. (2018) | * | * | * | * | - | * | * | * | 7 |
| Gaudet et al. (2019) | * | * | * | * | - | * | * | * | 7 |
| Gaudet et al. (2019) (2) | * | * | * | * | - | * | * | * | 7 |
| Gaudet et al. (2022) | * | * | * | * | - | * | * | * | 7 |
| Ying et al. (2019) | * | * | * | * | - | * | * | * | 7 |
| Whitney et al. (2020) | * | * | * | * | - | * | * | * | 7 |
| Yu et al. (2021) | * | * | * | * | - | * | * | * | 7 |
| Pascreau et al. (2021) | * | * | * | * | - | * | * | * | 7 |
| Levy et al. (2023) | * | * | * | * | - | * | * | * | 7 |
